# Off-Flavor Removal from Sheep Placenta via Fermentation with Novel Yeast Strain *Brettanomyces deamine* kh3 Isolated from Traditional Apple Vinegar

**DOI:** 10.3390/molecules26195835

**Published:** 2021-09-26

**Authors:** Han-Sol Choi, Keum-Yun Ha, Xing-Yue Xu, Hee-Cheol Kang, Hoon Kim, Yeon-Ju Kim

**Affiliations:** 1 Graduate School of Biotechnology, College of Life Science, Kyung Hee University, Yongin 17104, Korea; chsol96@khu.ac.kr (H.-S.C.); hispoon@khu.ac.kr (K.-Y.H.); xingyue5125@khu.ac.kr (X.-Y.X.); 2 GFC Life Science Co. Ltd., F17, Apexcity, 823, Dongtansunhwan-daero, Hwasung 18471, Korea; michael@gfcos.co.kr; 3 College of Biotechnology and Natural Resources, Chung-Ang University, Anseong 17546, Korea

**Keywords:** sheep placenta, off-flavor, deodorization, *Brettanomyces* sp, fermentation, sensory evaluation

## Abstract

Animal placentae can be used as health-promoting food ingredients with various therapeutic efficacies, but their use is limited by their unpleasant odor and taste. This study aimed to investigate the possibility of deodorization of sheep placenta via yeast fermentation. A yeast strain was successfully isolated and identified as a novel *Brettanomyces* strain (*Brettanomyces deamine* kh3). The deodorizing efficacy of fermentation of the sheep placenta with *B. deamine* kh3 was evaluated by 42 panels, based on evaluation of preference, ranking, and aroma profiles, and compared with normal placenta and placenta fermented with *B. bruxellensis*. The results of the sensory evaluation indicated that fermentation of the sheep placenta with *B. deamine* kh3 may improve its palatability by increasing flavors such as that of grass (tree), rubber, and burnt, and by decreasing the odor and soy sauce flavor. Solid-phase microextraction-gas chromatography (SPME-GC) showed that major off-flavors in sheep placenta, such as ammonia, dimethyl disulfide, and 1,3-dioxolane, were completely diminished in the sheep placenta fermented with *B. deamine* kh3. This study presents those major volatile compounds, including 2-isobutyl\-4,4-dimethyl-1,3-dioxane, and 3-methyl-1-butanol, could be crucial in improving the palatability of the sheep placentae fermented with *B. deamine* kh3. This study provides a good starting point for the industrial application of a new deodorization method.

## 1. Introduction

The placenta is a temporary organ, originating from the embryos during pregnancy, and is physiologically expelled during childbirth [1]. It is a highly specialized organ that is crucial for providing nutrition and protection to the fetus by exchanging nutrients, oxygen, gases, and antibodies with the mother [2,3]. In recent years, the postpartum placenta emitted after childbirth has been shown to be eaten in some cultures, owing to the belief that it is beneficial for human health, a practice which is now defined as placentophagy [4]. Moreover, the human placenta has been considered a traditional medicine for stimulating wound treatment and tissue regeneration. Current studies indicate that the human placenta has various therapeutic effects, such as antioxidant [5], antimicrobial [6], anti-inflammatory [7,8], anti-allergy [9], anti-fatigue [10], liver protection [11], and immunomodulation [12] effects. Although it has a wide range of therapeutic activities, the human placenta is limited in its wide industrial applications owing to ethical considerations. Thus, placentae from animals such as sheep, goats, horses, pigs, and cattle have attracted attention as alternatives to the human placenta [2]. In particular, sheep placentae are emerging in the cosmetic industry as cosmeceuticals that can improve skin health and treat skin diseases. Although there are important differences in the structure and function of different groups of mammals, there are few significant differences in the biochemical contents of the placental tissue across various species [4]. The placenta contains many biologically active ingredients, such as enzymes, amino acids, peptides, polydeoxyribonucleotides, vitamins, growth factors, and trace elements [13]. Conversely, the placenta has a distinctively metallic odor and an unpleasant taste that is difficult to describe but is easily recognized with experience [14]. The unpleasant odors emitted by the animal placenta are considered a major hurdle in expanding its wide industrial applications, and the flavor and taste of products are crucial factors for consumer acceptability of the finished product. Unfortunately, no study has reported the chemical elucidation of the main contributors to the undesirable flavors in the placenta. To our knowledge, only the encapsulation method has been widely used for placentophagy to mask the off-flavors of placenta [14], but few studies have investigated the deodorization of the off-flavor of animal placenta.

Traditionally, various deodorization techniques in the agricultural and environmental industries have been based on three technical categories: chemical, physical, and biological processes [15]. Compared with the first two processes, biological deodorization has environmental and economic advantages, and it is thus considered a more robust and reliable alternative to conventional physical or chemical processes [16]. Recently, the application of fermentation, a biological process that produces chemical changes in organic compounds via the metabolic processes of microorganisms, has gained attention for the reduction in off-flavors in various natural substances [17,18]. Various microbial strains, such as lactic acid bacteria [19] and fungi [17,20] are currently used to remove off-flavors from various natural substances. However, few studies have investigated the deodorization of natural substances, including animal placentas, via biological processes using yeast fermentation. The Ascomycetous genus *Brettanomyces*, belonging to the family *Pichiaceae*, is also interchangeably called the genus *Dekkera*. Scientifically, the use of the terms *Brettanomyces* and *Dekkera* is known based on its sexual forms (anamorphic and teleomorphic, respectively) [21]. The *Brettanomyces* genus was first isolated in 1903 by Hjelte Clausen, of the Carlsberg Research Laboratory, and later became known as *Brettanomyces clausenii* [21,22]. This genus has characteristics such as low pH resistance, unique flavor production, and high ethanol yield [21,22]. The genus *Brettanomyces* is considered an unfavorable yeast because of its spoilage property that produces off-flavors in various foods, such as wine, beer, ciders, daily products, and soft drinks [23]. However, a recent study reported that *Brettanomyces* species can produce fresh exotic flavors, suggesting their use in such applications.

Therefore, this study aimed to investigate the possibility of deodorization of sheep placenta via yeast fermentation. In this study, a novel yeast strain was recently isolated from apple vinegar, and its genetic characteristics were investigated via internal transcribed spacer (ITS) region sequencing. In addition, various fermentation conditions, using the novel yeast for the removal of off-flavors in the sheep placenta, were investigated via sensory evaluation. In addition, solid-phase microextraction-gas chromatography (SPME-GC) analysis was employed for comparative determination of flavor compounds between normal and fermented placentas with yeast.

## 2. Results and Discussion

### 2.1. Isolation and Identification of B. deamine kh3

**Figure 1:**
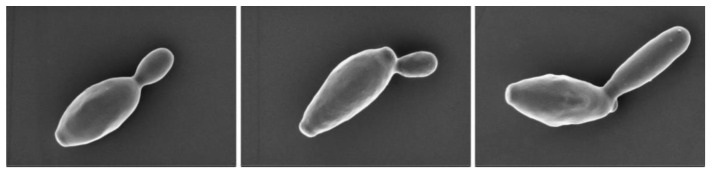
Scanning electron microscopy (SEM) images of *Brettanomyces deamine* kh3.

A novel *Brettanomyces* species, *B. deamine* kh3 was successfully isolated from apple vinegar and registered as a novel *Brettanomyces* genus and granted accession numbers from the Korean Collection for Type Cultures (KCTC; 14262BP) and the National Center for Biotechnology Information (NCBI; MT755882). *Brettanomyces* has recently been applied in the production of craft, specialty beers, and wines [23]. Subsequently, we investigated the possibility of off-flavor removal, using the novel isolated *Brettanomyces* species, *B. deamine* kh3. The morphological characteristics of *B. deamine* kh3 were observed using scanning electron microscopy (SEM). The SEM images indicated that the morphology of *B. deamine* kh3 exhibited a rod-shaped and round, short length of approximately 4.8 µm (Figure 1), which is commonly observed in yeast strains.

**Figure 2:**
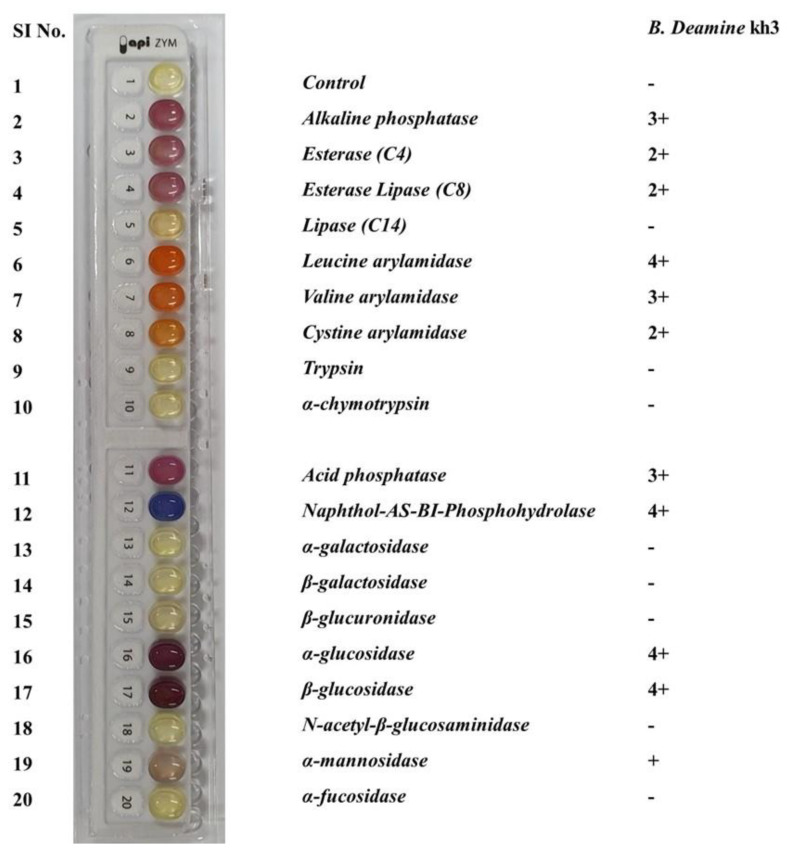
Enzymatic activity profile of *B. deamine* kh3 using a commercial assay kit. Different color developments show the activity of various enzymes with varying degrees of intensity. The *B. deamine* kh3 has positive activity for 11 enzymes, which were grouped as strong (from 3 to 5+) or weak (from 1 to 2+) based on the intensity of color development in their respective cupules.

The physiological properties of the novel *B. deamine* kh3 strain were evaluated in terms of optimal growth conditions (temperature, pH, NaCl concentration, and growth medium) and characteristics (enzymatic activity and tolerance test) (Supplementary Table S2). Results indicated that the strain could grow at temperatures of 20–40 °C (optimum 30 °C), pH of 4.0–10.0 (optimum 7.0), NaCl concentration under 7%, and ethanol concentration less than 8%. Upon monitoring the substrate degradation activity, the strain could hydrolyze esculin, but it did not work for gelatin, casein, DMA, Tween 20, and Tween 80. The strain was found to have growth capability under certain growth media, such as yeast malt (YM) agar, yeast extract peptone dextrose (YPD) agar, trypticase soy agar (TSA), nutrient agar (NA), Luria-Bertani (LB) agar, potato dextrose agar (PDA), De Man, Rogosa, and Sharpe (MRS) agar, Czapek yeast extract agar (CYA), Reasoner’s 2A (R2A) agar, and MacConkey (MAC) agar (data not shown). Furthermore, the enzymatic activity profiles of our novel strains were obtained using a commercial API-ZYM kit assay. The results showed that *B. deamine* kh3 showed positive activity against 11 enzymes. Among them, the strongest activity (3 to 5+) was observed for alkaline phosphatase, leucine arylamidase, valine arylamidase, acid phosphatase, naphthol-AS-BI-phosphohydrolase, α-glucosidase, and β-glucosidase. However, a relatively lower activity (1 to +2) was observed for esterase (C4), esterase lipase (C8), cystine arylamidase, and α-mannosidase. No visible activity was observed for lipase (C14), trypsin, α-chymotrypsin, α-galactosidase, β-galactosidase, β-glucuronidase, *N*-acetyl-β-glucosaminidase, and α-fucosidase (Figure 2). 

**Figure 3:**
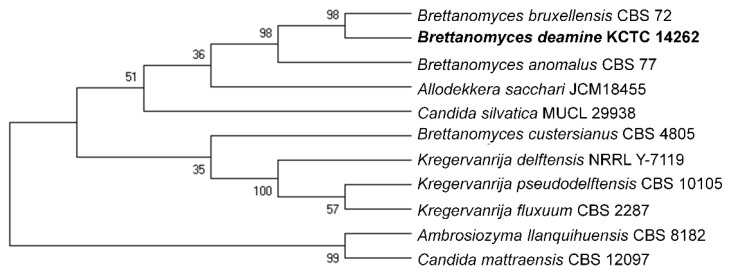
Statistical method of neighbor-joining tree, based on the ITS region sequences of *Brettanomyces deamine* kh3 (bold letter); Rooted neighbor-joining tree, based on the ITS region sequences, shows the phylogenetic position of the yeast strain, *B. deamine* kh3, and the other closest yeast genus, *Brettanomyces*, as well as some other related taxa.

As can be observed in the ITS phylogenetic tree (Figure 3), the closest relative of *B. deamine* kh3 was *B. bruxellensis* with 95.5% identity, which was formed in the same clade.

### 2.2. Establishment of Optimal Method for the Fermented Sheep Placenta

**Figure 4:**
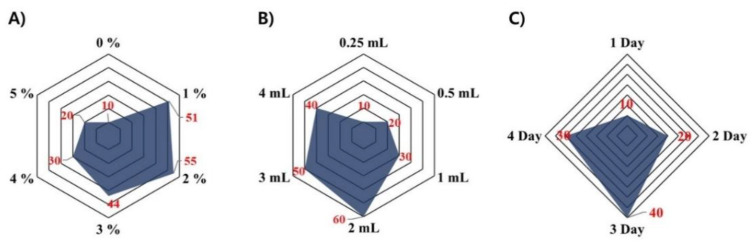
Quantitative descriptive sensory analysis, using 10 untrained volunteers, for establishing optimal process of the sheep placenta, fermented with *B. deamine* kh3, depending on (**A**) glycerol concentration, (**B**) volume of yeast suspension, and (**C**) fermentation period. The intensity evaluation of the sensory perception was performed by selecting structured scales of 0 (the worst smell) to 4 or 6 (the best smell; depending on the number of testing factors) at an interval of 1. Results were expressed as sum of individual score.

To investigate the possibility of deodorization of the sheep placenta, the sheep placentae were fermented with *B. deamine* kh3, varying the concentration of the carbon source (glycerol concentration), volume of yeast cell suspension, and fermentation period. Fermented sheep placentae were evaluated by sensory testing of 10 untrained volunteers. As shown in Figure 4A–C, the optimal conditions for preparing the fermented sheep placenta were as follows: 2% glycerol concentration, 2 mL *B. deamine* kh3 suspension, and fermentation for 3 days. The optimal fermentation method was used for further studies on the preparation of fermented sheep placentas.

### 2.3. Preference and Ranking Determination via Sensory Evaluation

Sheep placentae fermented with *B. deamine* kh3 were prepared under optimal conditions. Thereafter, its deodorizing efficacy was evaluated by comparing to the normal sheep placenta and sheep placenta fermented with *B. bruxellensis*. The sensory test groups were evaluated by sensory testing of 42 panels and are listed in Table 1 and Supplementary Table S1.

The results are expressed as preference and rank scales (Table 2 and Table 3, respectively). The preference test was conducted on a scale of 0 (bad smell) to 10 (good smell) at intervals of 2. According to the results of the preference test (Table 2), the average scores of normal sheep placenta, *B. deamine* kh3, sheep placenta fermented with *B. deamine* kh3, and sheep placenta fermented with *B. bruxellensis* were 4.48, 5.71, 5.33, and 4.57, respectively. As expected, the normal sheep placenta (SP) and sheep placenta fermented with *B. deamine* kh3 (SP-BD) received the lowest and highest scores, respectively, and a statistically significant difference (*p* < 0.05) was observed between the two samples (Table 2).

Compared with the sheep placenta-tested group (4.48), the group testing the sheep placenta fermented with *B. bruxellensis* (4.57) did not exhibit a significant difference (*p* > 0.05), whereas that of sheep placenta fermented with *B. deamine* kh3 (5.33) exhibited a significantly higher score (*p* < 0.05). This indicated that the low preference for normal sheep placenta could be improved by fermentation with *B. deamine* kh3. Thereafter, a ranking test was performed on three samples: one sheep placenta and two fermented sheep placentae (Table 3). The ranking test was performed by selecting the worst, normal, and best samples by marking numbers 1, 2, and 3, respectively. The test group of the normal sheep placenta was selected as the worst, medium, and best smells from 15, 13, and 14 individuals, respectively. Sheep placentas fermented with *B. deamine* kh3 were selected as the worst, medium, and best smells from 10, 14, and 18 individuals, respectively. Sheep placentas fermented with *B. bruxellensis* were selected as the worst, medium, and best smells from 17, 15, and 10 individuals, respectively. The average scores of groups tested with the normal sheep placenta, placenta fermented with *B. deamine* kh3, and placenta fermented with *B. bruxellensis* were 1.98, 2.19, and 1.83, respectively. Although the two groups tested with fermented sheep placentae did not exhibit statistically significant differences (*p* > 0.05) compared with the normal sheep placenta, based on the average score, the group tested with the sheep placenta fermented with *B. deamine* kh3 showed the most favorable result compared with groups tested with the sheep placenta or the sheep placenta fermented with *B. bruxellensis*.

### 2.4. Determination of Aroma Profile via Sensory Attribute

**Figure 5:**
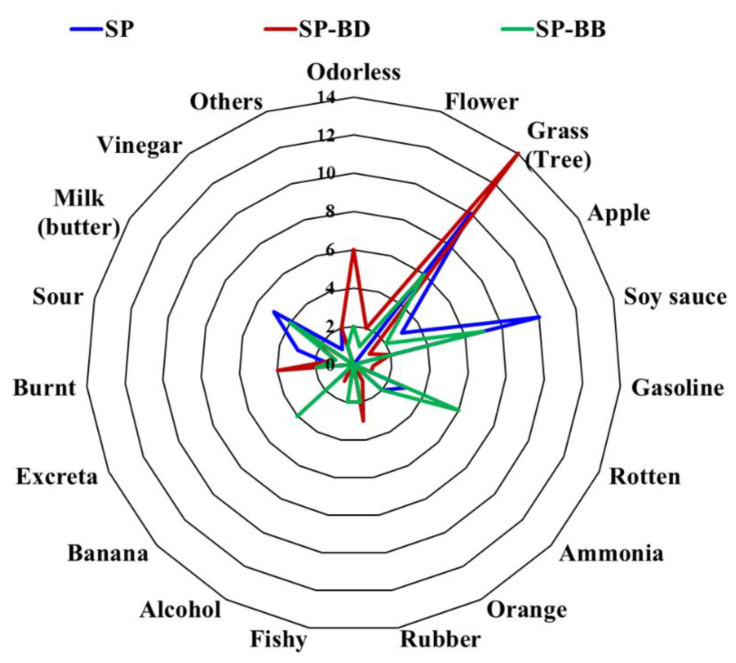
Quantitative descriptive sensory analysis, using 42 panels, for evaluating specific flavors. Results of the sensory perception were filled in with the questionnaire provided. SP, sheep placenta; SP-BD, sheep placenta fermented with *B. deamine* kh3; SP-BD, sheep placenta fermented with *B. bruxellensis*.

The typical aroma profiles generated from each sample were surveyed: odorless, flower, grass (tree), apple, soy sauce, gasoline, rotten, ammonia, orange, rubber, fishy, alcohol, banana, excreta, burnt, sour, milk (butter), vinegar, and others. As shown in Figure 5, panels selected grass (tree) (23.8%) and soy sauce (23.8%) smells as the major aromas for the sheep placenta, whereas grass (tree) (33.3%) and odorless (14.3%) smells were selected as the major aromas for the placenta fermented with *B. deamine* kh3. Soy sauce (16.7%), tree (14.3%), and rotten (14.3%) smells were the major aromas of sheep placenta fermented with *B. bruxellensis*. Interestingly, odorless (+14.3%), grass (tree) (+9.5%), rubber (+7.1%), and burnt (+7.1%) smells increased, whereas the score of soy sauce (−19.0%) drastically decreased for the sheep placenta fermented with *B. deamine* kh3, compared with the normal sheep placenta. This indicates that fermentation of sheep placenta with *B. deamine* kh3 could improve its flavor preference by changing its major aromas.

### 2.5. Determination and Identification of Volatile Compounds in the Fermented Placenta via SPME-GC

SPME-GC analysis was performed to monitor and compare the modification of major volatile compounds in the normal sheep placenta and the placenta fermented with *B. deamine* kh3. As shown in Table 4, the normal sheep placenta contained ammonia (16.7%), dimethyl disulfide (13.5%), 4-methyl-2-heptanone (11.7%), 2-hexanol (11.6%), hexane (11.0%), acetone (10.5%), and 1,3-dioxolane (10.4%) as the major volatile compounds. In particular, the presence of well-known unpleasant odors, including ammonia [24], 4-methyl-2-heptanone [25], dimethyl disulfide [26], 1,3-dioxolane [27], and organic solvents, such as hexane and acetone, could affect the odors of the sheep placenta. Compared with the normal sheep placenta, major off-flavors such as ammonia, dimethyl disulfide, and 1,3-dioxolane were completely diminished in the sheep placenta fermented with *B. deamine* kh3. In contrast, a large quantity of the little-known compound, 2-isobutyl-4,4-dimethyl-1,3-dioxane, was generated in the sheep placenta fermented with *B. deamine* kh3. Unfortunately, this study could not clearly demonstrate that the unknown compound originated from the fermentative characteristics of the fermented sheep placenta. To our knowledge, few studies on the physicochemical properties of 2-isobutyl-4,4-dimethyl-1,3-dioxane have been reported. Thus, further studies are necessary to characterize the physicochemical properties through the separation and purification of 2-isobutyl-4,4-dimethyl-1,3-dioxane in the near future. Moreover, an alcoholic compound, 3-methyl-1-butanol (isoamyl alcohol), was produced in sheep placenta fermented with *B. deamine* kh3. Notably, 3-methyl-1-butanol is a key volatile compound responsible for whiskey, malt, and burned aromas in various types of wine [28,29]. The results indicate that major compounds, such as 2-isobutyl-4,4-dimethyl-1,3-dioxane and 3-methyl-1-butanol, may play important roles in improving the preference of sheep placenta fermented with *B. deamine* kh3. Furthermore, our study provides a good starting point for the industrial application of a new deodorization method using a novel yeast strain, *B. deamine* kh3.

## 3. Materials and Methods

### 3.1. Reagents

Commercially hydrolyzed and freeze-dried sheep placenta powder (Cat. No. 65155) was purchased from Starboard Bio Ltd. (Hawke Bay, New Zealand). The growth media, such as YM agar, YPD agar, TSA, NA, LB agar, PDA, MRS agar, CYA, R2A agar, and MAC agar, were supplied by Kisan Bio (Seoul, Korea). The glycerol, gelatin, casein, esculin, Tween 20, and Tween 80 were purchased from Sigma-Aldrich (St. Louis, MO, USA). The *B. bruxellensis* strain was obtained from the Korean Culture Center of Microorganisms (KCCM 19490; Seoul, Korea).

### 3.2. Isolation and Identification of the Novel Yeast Strain from Fermented Apple Vinegar

The novel strain was isolated from traditionally fermented apple vinegar in Yongin Province (Gyeonggi, Korea). The stock solution of vinegar was diluted one in ten with sterilized saline (Seoul, Korea), followed by serial dilution to 10^−6^. Each solution was plated onto YPD agar plates, using the general streak plate method, and then incubated in an incubator at 25 °C for 48 h. After incubation, major colonies were selected based on colony morphology, and were subsequently transferred and separated from the colonies of other microorganisms on the agar. The isolated strain was diluted in YPD broth medium, containing 30% (*v*/*v*) glycerol, and stored at −80 °C for future study. To identify the yeast strain, ITS region sequences were analyzed using Genocell (Daejeon, Korea). The associated taxa were searched using the GenBank database at the National Center for Biotechnology Information (NCBI; https://www.ncbi.nlm.nih.gov/taxonomy, accessed on 23 August 2021), and multiple alignments from the related taxa database were organized using cluster software. Phylogenetic trees were constructed with the bootstrap test (1000 replicates) in the Molecular Evolutionary Genetics Analysis (Mega)-X website (https://www.megasoftware.net, accessed on 23 August 2021) using the neighbor-joining method [30].

### 3.3. Evaluation of Physiological Characteristics and Enzymatic Activity of the Novel Yeast Strain

The growth capability of *B. deamine* kh3 was investigated with various growth media, including YPD agar, NA, TSA, PDA, MRS agar, LB agar, CYA, YM agar, MAC agar, and R2A agar. The optimal temperature for *B. deamine* kh3 growth were monitored on YPD agar plate at temperatures of 4, 10, 20, 25, 30, 37, 40, and 42 °C for 7 days incubation. The growth in a range of pH values was examined under pH values, ranging from 4.0 to 10.0 with 0.5 pH unit intervals, in YPD broth adjusted with citric acid/sodium citrate (pH 4.0–6.0), Na_2_HPO_4_/NaH_2_PO_4_ (pH 6.0–8.0), Na_2_CO_3_/NaHCO_3_ (pH 8.0–10.0), and Na_2_HPO_4_/NaOH (pH 10.0) at 30 °C for 7 days. The substrate degradation activity was evaluated on YPD agar plate containing esculin, gelatin, casein, DMA, Tween 20, or Tween 80 for 7 days at 30 °C. The effect of *B. deamine* kh3 on tolerance against salinity and alcohol was estimated in YPD broth, containing NaCl concentrations ranging from 0 to 10% with 1% intervals, and containing ethanol concentrations, ranging from 0 to 10% with 1% intervals, respectively [31]. The activity of 19 enzymes in *B. deamine* kh3 was examined using a commercial API-ZYM kit (Bio-Merieux, Lyon, France) following the manufacturer’s instructions. In brief, *B. deamine* kh3 was diluted to 5 McFarland turbidity in distilled water. This *B. deamine* kh3 suspension was inoculated into each cupule in the API-ZYM strip at a level of 65 μL and incubated at 37 °C for 4.5 h. The reaction result was determined from the color produced following incubation for 5 min under bright light after adding ZYM-A and ZYM-B reagents to each cupule. The results were recorded according to the APY-ZYM color reaction chart, using a scale from 0 to 5+, based on the visual color intensity [32].

### 3.4. Establishment of Optimal Condition for Fermentation of Sheep Placenta

The *B. deamine* kh3 strain was washed twice with sterilized distilled water and diluted with sterilized saline solution to adjust the cell number to 3 × 10^7^ colony forming units (CFU)/mL (absorbance at 600 nm = 1.0). The absorbance was determined using a UV-Vis spectrophotometer (Genesys 6; Thermo Fisher Scientific, Waltham, MA, USA) and absorption cuvettes (100-QS; Hellma, Muellheim, Germany). Sheep placenta solution (1%, *w*/*v*) was prepared by dissolving sheep placenta powder in sterilized diluted water, and sterilization was performed using an autoclave machine (BF-45AC; Biofree Co., Ltd., Seoul, Korea). The diluted *B. deamine* kh3 suspension was added to a solution containing the sheep placenta solution and glycerol, and then, it was incubated in a shaking incubator (VS-8480S; Vision Scientific Co., Ltd., Daejeon, Korea) at 30 °C. To optimize fermentation, various ferments were prepared based on glycerol concentration, cell density of *B. deamine* kh3, and fermentation time, and were then evaluated via quantitative descriptive sensory analysis (QDA) using 10 untrained volunteers (undergraduate and graduate students at Kyunghee University; two males and eight females). To establish the optimal conditions for fermentation, the glycerol concentration was first investigated, and *B. deamine* kh3 density and fermentation time were optimized. QDA was carried out in individual booths at temperatures of 20–24 °C and a relative humidity of 40% under daylight. Testing samples were presented in lidded containers, labeled with randomly selected 3-digit numbers, on the table. The intensity evaluation of the sensory perception was performed by selecting structured scales of 0 (the worst smell) to 4 or 6 (the best smell; depending on the number of testing factors) at an interval of 1.

### 3.5. Sensory Evaluation

QDA was performed by a panel of 42 participants, consisting of one male and 41 females at the Korea Dermatology Research Institute (KDRI; Seongnam, Korea). The sensory evaluation was approved by the KDRI’s Institutional Review Board (KDRI-IRB-20496) and was performed in accordance with the tenets of the Helsinki declaration [33], which protects the rights and interests of the subjects, and complies with human test management standards, as well as domestic regulations, in conducting research and recording results. Detailed information on the panel is provided in Supplementary Table S1. During the evaluation, external odor inflow was blocked by an air-circulating system, and communication between the different groups was blocked in a partitioned area to prevent any interference. Testing samples were presented in plastic containers labeled with randomly selected 3-digit numbers on the table, and QDA was conducted by sniffing through wafting at a distance of approximately 10 cm from the sample. The sensory perception results were filled with a questionnaire. After the sensory evaluation of the prior sample, the panels were allowed to smell the prepared coffee beans and rest for one min to relieve fatigue of the nose. For items that answered the preferences and properties using preference and ranking scale methods, the mean value was calculated and statistical analysis (PASW Statistics 18; IBM Co., Armonk, NY, USA) was conducted using the Kruskal-Wallis test.

### 3.6. SPME-GC Analysis

SPME-GC [34] was used to measure and identify the volatile compounds in the sample solution. The volatile compounds were collected using SPEM fiber (mixing fiber of divinylbenzene/carboxen/polydimethylsiloxane (DVB/CAR/PDMS)] and then analyzed using a GC-MS system (HP 6890 GC/HP5973 MSD/multi-purpose sampler MPS2; Agilent, Santa Clara, CA, USA) equipped with a Hewlett Packard 19091X-133 HP-Wax capillary column (30 m × 25 mm, 25 μm; Hewlett Packard Enterprise, Spring, TX, USA). The temperature of the oven was set at 40 °C for 5 min, subsequently increasing 40 °C→180 °C (5 °C/min), and then held at 180 °C for 20 min. Ultra-high purity helium was used as the carrier gas at a flow rate of 1.0 mL/min. The injector and detector temperatures were set at 250 °C, and the temperature of the oven was set at 40 °C for 5 min, 40 °C→180 °C (5 °C/min), and held at 180 °C for 20 min. One microliter of sample solution was injected in split mode (20:1). The Scan/SIM mode was set at *m*/*z* 10–500. The data-dependent MS^2^ experiments were controlled using menu-driven in-house software, and the identification of volatile compounds was performed by comparing the MS^2^ spectrum in the Wiley 8.0 database. Compounds with peak areas below 18.5% in the GC-MS chromatogram were considered as not detected (n.d.).

### 3.7. Statistical Analysis

Statistical analysis (PASW Statistics 18; IBM Co., Armonk, NY, USA) was conducted using either Student’s *t*-test or Duncan’s multiple range test at a value of *p* > 0.05. *p* < 0.05, *p* < 0.01, and *p* < 0.001 were considered to indicate significant differences.

## 4. Conclusions

Although various physiological activities of animal placentae have been reported to date, their unfavorable flavor is a challenge that must be addressed to allow their widespread use. To perform the off-flavor removal process of sheep placenta, a novel yeast strain was successfully isolated from traditionally fermented apple vinegar. The yeast strain, *B. deamine* kh3, was used in the fermentation of the sheep placenta, and the efficacy of reducing off-flavors was tested by sensory evaluation. In this study, the sheep placenta fermented with *B. deamine* kh3 exhibited significantly better palatability, due to the reduction in off-flavors, compared with sheep placenta fermented with *B. bruxellensis* or the normal sheep placenta. Although the flavor preference increased with respect to sheep placenta fermented with *B. deamine* kh3 compared to the normal sheep placenta, a significant difference was not observed between the two groups in the sensory test using the ranking scale method. However, the preference for sheep placenta fermented with *B. deamine* kh3 was significantly higher than that of normal sheep placenta in the sensory test using the preference scale method. Specifically, when the sheep placenta was fermented with the novel yeast strain, *B. deamine* kh3, changes were observed in various volatile compounds (ammonia, dimethyl disulfide, 1,3-dioxolane, and 2-isobutyl-4,4-dimethyl-1,3-dioxane), which could explain the deodorizing effect and increase in flavor preference. This study proposes a good starting point for the industrial application of a new deodorization method using the novel yeast strain, *B. deamine* kh3.

## Figures and Tables

**Table 1:** Information of testing group, for quantitative descriptive sensory analysis, using 42 panels.

Group	Sample Information	Sheep Placenta	Yeast Strain	Abbreviation
1	Sheep placenta	+	-	SP
2	Sheep placenta fermented with *B. deamine*	+	*B. deamine*	SP-BD
3	Sheep placenta fermented with *B. bruxellesis*	+	*B. bruxellesis*	SP-BB

Both *B. deamine kh3* and *B. bruxellensis* strains were washed with sterilized water and diluted with sterilized saline solution. The sheep placenta was individually fermented with *B. deamine* kh3 or *B. bruxellensis*, respectively, in the 2% glycerol-containing water for 3 days (SP-BD and SP-BB). In contrast, normal sheep placenta was dissolved with 2% glycerol in water, without a yeast strain, and incubated for the same period. SP, sheep placenta; SP-BD, sheep placenta fermented with *B. deamine* kh3; SP-BB, sheep placenta fermented with *B. bruxellensis.*

**Table 2:** Quantitative descriptive sensory analysis using the preference scale method.

Group	N	Mean	*Z*	*H*	*p*-Value	Rank
SP	42	4.48	−2.12	13.44	0.004	3
SP-BD	42	5.33 *	1.26			1
SP-BB	42	4.57	−1.77			2

QDA was evaluated using 42 panels and the preference test was investigated in the scale of 0 (bad smell) to 10 (good smell) at an interval of 2. SP, sheep placenta; SP-BD, sheep placenta fermented with *B. deamine* kh3; SP-BB, sheep placenta fermented with *B. bruxellensis*. Statistical analysis was carried out using Student’s *t*-test between SP group and each group. * *p* < 0.05.

**Table 3:** Quantitative descriptive sensory analysis using the ranking scale method.

Group	N	Mean	*Z*	*H*	*p* Value	Rank
SP	42	1.98	−0.22	4.04	0.133	2
SP-BD	42	2.19	1.74			1
SP-BB	42	1.83	−1.52			3

QDA was evaluated using 42 panels, and the ranking test was performed by selecting the worst, normal, and best samples by marking numbers 1, 2, and 3, respectively. SP, sheep placenta; SP-BD, sheep placenta fermented with B. deamine kh3; SP-BB, sheep placenta fermented with B. bruxellensis. Statistical analysis was carried out using Student’s *t*-test between SP and each group.

**Table 4:** Determination and identification of volatile compounds in the fermented placenta via SPME-GC analysis.

NO.	Chemical Compound	Formula	Area (%)	
			SP	SP-BD
1	Hexane	C_6_H_14_	11.0	12.1
2	Methylcyclopentane	C_6_H_12_	n.d.	7.8
3	1,3-dioxolane	C_3_H_6_O_2_	10.4	n.d.
4	4-Methylheptane	C_8_H_18_	n.d.	4.0
5	2,4-Dimethylheptane	C_9_H_20_	n.d.	2.6
6	Acetone	C_3_H_6_O	10.5	6.2
7	2,4-Dimethyl-1-heptene	C_9_H_18_	8.9	3.0
8	2-Butanone	C_4_H_8_O	2.8	n.d.
9	2-Pentanone	C_5_H_10_O	1.5	n.d.
10	Dimethyl disulfide	C_2_H_6_S_2_	13.5	n.d.
11	Ammonia	NH3	16.7	n.d.
12	2-Isobutyl-4,4-dimethyl-1,3-dioxan	C_10_H_20_O_2_	n.d.	26.0
13	Decamethyl-cyclopentasiloxane	C_10_H_30_O_5_Si_5_	0.5	n.d.
14	4-Methyl-2-heptanone	C_8_H_16_O	11.7	1.1
15	3-Methyl-1-butanol	C_5_H_12_O	n.d.	16.6
16	2-Hexanol	C_6_H_14_O	11.6	n.d.
17	α-Methyl-β-oxo-2-pyridinepropanoic acid ethyl ester	C_9_H_9_NO_3_	1.3	n.d.
18	4,6-Dimethyl-2-heptanone	C_9_H_18_O	1.6	1.3
19	1,3-Bis(1,1-dimethylethyl) benzene	C_14_H_22_	2.7	3.5
20	Benzene-ethanol	C_8_H_12_O	n.d.	7.5
21	Trace materials	-	6.3	6.6

Various volatile compounds were determined using SPME-GC analysis, and area (%) was calculated as percentage of area of target compounds against the area of total compounds shown in the GC-MS chromatogram. SP, sheep placenta; SP-BD, sheep placenta fermented with *B. deamine* kh3; n.d., not determined.

## Data Availability

No new data were created or analyzed in this study. Data sharing is not applicable to this article.

## Supplementary Materials

The following are available online, Table S1: Information of 42 panels who participated in QDA of this study, Table S2: Physiological characteristics of *B.deamine* kh3.

## Sample Availability

Samples of the sheep placenta fermented with *B. deamine* kh3 are available from the authors.
