# Supplementary material for: Off-Flavor Removal from Sheep Placenta via Fermentation with Novel Yeast Strain Brettanomyces deamine kh3 Isolated from Traditional Apple Vinegar
Source: Molecules. 2021 Sep 26;26(19):5835. doi: 10.3390/molecules26195835 (PMC8510316; doi:10.3390/molecules26195835)
Supplement: Supplementary file 1 [file molecules-26-05835-s001.zip › molecules-1282344-supplementary.pdf]

NO.	ID	Age	Gender	NO.	ID	Age	Gender
1	4693	20	Female	22	2740	47	Female
2	3487	21	Female	23	4293	47	Female
3	4078	22	Female	24	3794	47	Female
4	4870	22	Female	25	1719	48	Female
5	4248	23	Female	26	2909	48	Female
6	2682	23	Female	27	4326	49	Female
7	3475	25	Male	28	4633	50	Female
8	5005	35	Female	29	3201	50	Female
9	4829	39	Female	30	3512	50	Female
10	3705	39	Female	31	3528	50	Female
11	2069	42	Female	32	3697	52	Female
12	2379	43	Female	33	4303	52	Female
13	3935	43	Female	34	3265	52	Female
14	3831	43	Female	35	3730	52	Female
15	2075	43	Female	36	2295	53	Female
16	2528	45	Female	37	5084	53	Female
17	2984	46	Female	38	2922	53	Female
18	2771	46	Female	39	3478	53	Female
19	2987	46	Female	40	3883	54	Female
20	1642	47	Female	41	2450	56	Female
21	5083	47	Female	42	2155	58	Female

Supplementary Table S1. Information of 42 panels who participated in QDA of this study.

**Physiological characteristic**	**Tested (optimal or applicable)**
Growth range	
Temperature (°C)	20 to 40 (30)
pH	4.0 to 10.0 (7.0)
NaCl concentration (%)	0 to 10 (≤7)
Ethanol concentration (%)	0 to 10 (≤8)
Substrate degradation activity	
Gelatin	–
Esculin	+
Casein	–
DNA	–
Tween 20	–
Tween 80	–

Supplementary Table S2. Physiological characteristics of *B. deamine* kh3.
